# Testing performance of large-scale surveys in determining trends for the critically endangered Great Indian Bustard *Ardeotis nigriceps*

**DOI:** 10.1038/s41598-019-48193-2

**Published:** 2019-08-12

**Authors:** Shaheer Khan, Nilanjan Chatterjee, Bilal Habib

**Affiliations:** Wildlife Institute of India, Dehradun, India

**Keywords:** Conservation biology, Population dynamics

## Abstract

Great Indian Bustard (GIB) is listed as Critically Endangered, with less than 250 individuals surviving in three fragmented populations. The species is under tremendous threat due to various anthropogenic pressures. Effective management and conservation of GIB requires a proper monitoring protocol, which we propose using an occupancy framework approach to detect changes in the species’ population. We used occupancy estimates from various landscape level surveys and simulated scenarios to evaluate the effectiveness of the proposed protocol. Our result showed there is >70% chance of detecting 100% change in the occupancy with 100 sampling sites and 10 temporal replicates. While with double sampling sites, the same change can be detected with 4–6 temporal replicates. In absence of a robust population estimation method, we argue for the use of occupancy as a surrogate to detect change in population as it provides better insights for rare elusive species such as GIB. Our proposed methodological framework is more precise than previous methods, which will help in evaluating efficacy of management interventions proposed and the implementation of species recovery plans.

## Introduction

Great Indian Bustard (GIB), India’s heaviest flying bird, was once abundant in dry grasslands of India. It is now categorized as Critically Endangered^1^ with less than 250 individuals surviving in three fragmented populations. The species is threatened by habitat loss, due to the conversion of grasslands into agriculture, transmission lines, wind turbines, feral dogs, livestock grazing intensification and hunting. As a result, its population has declined by 80% in 50 years from 1260 to 250 individuals^2,3^. This decline in GIB numbers has been recorded from all regions of its present distribution range^4^. Many site-specific targeted interventions, such as fencing of breeding sites, control of dog populations, and *ex-situ* conservation, have been planned to save the species from extinction^4^. In order to evaluate the efficacy of these interventions, the monitoring methods should be robust enough to detect any increase or decrease in GIB population size. The previous landscape level population estimation surveys across different monitoring years were done using a protocol developed by Dutta *et al*.^5^. This protocol has given results with a broad range of estimates which were 41–165 individuals in 2014^5^ and 92–240 individuals in 2016^6^. This broad range estimation interval hinders inference of population trend which impedes the process of testing the efficiency of management interventions.

Long-term conservation effort for any species relies on a sound understanding of its ecology, along with landscape-level population estimation^7^. Moreover, robust population estimation is fundamental as it provides an insight into the current status which helps in improving management policies^8–11^. Owing to the nomadic behaviour, long-life span, isolated population, rarity and long-ranging behavior^12^ of the GIB, it is very difficult to infer population trends using existing survey protocol and the reasons therein to serve as a rationale for conservation actions. Species with such natural history characteristics create unique challenges for both the design and implementation of population estimation surveys^13^.

The broad range of population estimates acquired using distance sampling from previous surveys (41–165 birds and 92–240 birds) can be made precise by increasing the sampling effort, but species behaviour like lekking may influence the detection probability in the subsequent visits. Therefore, it is logical to examine the change in habitat occupancy of the species rather than using distance sampling to infer change in numbers^14,15^. Occupancy modelling is based on presence and absence data and is most suited for population monitoring programs of rare and elusive species. Occupancy provides reliable information about species range, the probability of extinction^16^, meta-population dynamics^17^ and can be used as a surrogate of abundance^18^. Moreover, occupancy data are considered more effective for the study of rare species where abundance estimation is logistically and financially challenging^14,19^.

Under such circumstances, the need is to design a survey protocol which is logistically viable and able to detect change in number or occupancy within a given time frame. The statistical power to infer change in site occupancy is low with expedient number of temporal and spatial replicates^20,21^. In order to detect any change in GIB occupancy, we simulated different combinations of number of replicates and sites to reach the “acceptable” statistical power (typically 0.8) with a robust and feasible sampling design.

The aim of our study was to evaluate the effectiveness of the existing monitoring protocol to infer any change in the occupied area using both detection probability and occupancy estimates of previous surveys. We simulated detection histories with estimated parameters to evaluate the robustness of the surveys with 80% confidence interval. The outcome of the study will help in evaluating the implementation of target-oriented management plans to save the species from extinction threat and to guide future management interventions for conservation of endangered species.

## Results

In Rajasthan, a total of 118 grids (12 × 12 km) covering 16,992 km^2^ were surveyed along 1924 km (mean 16 ± 4 km) of transects in 2014^5^. In 2016, a total of 120 grids covering 17,280 km^2^ were surveyed along 2,273 km of transects^6^. In Maharashtra, a total of 372 grids covering 53,568 km^2^ were surveyed along 6436.6 km (mean 3.03 ± 1.74 km) length of transect for GIB population estimation in 2017^12^. The occupancy and detection probability estimates of live GIB of Rajasthan were 0.25 and 6.66% and 0.52 and 11% in 2014 and 2016, respectively^5,6^. The detection probability and occupancy of 30 dummy GIB in the sampling grids of Maharashtra were found to be 0.13 and 8.06% (Table 1)^12^.

**Table 1:** Details of the survey conducted in the state of Rajasthan and Maharashtra for GIB in 2014, 2016 and 2017 respectively.

Survey details	Number of grids surveyed	Area surveyed (sq. km)	Estimated occupancy (ψ)	Detection probability (p)
Rajasthan State, 2014	118	16,992	0.06	0.25
Rajasthan State, 2016	120	17,280	0.11	0.52
Maharashtra State, 2017^*^ (Blind test)	372	53,568	0.08	0.13

Estimates of occupancy and detection probability are given in the table.

^*^Calculated using life-size dummy to test the detection probability and occupancy.

**Figure 1:**
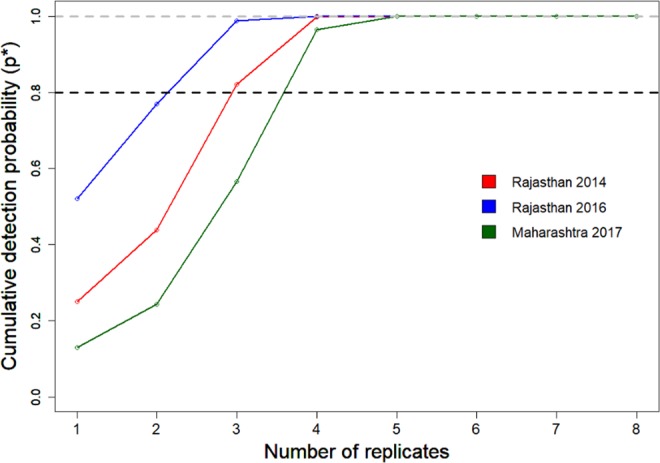
Effect of a number of replicates on the detection probability of the GIB surveys. All the surveys were carried out with one replicate though the cut-off of 0.8 is crossed with 3–4 replicates for all the surveys.

**Figure 2:**
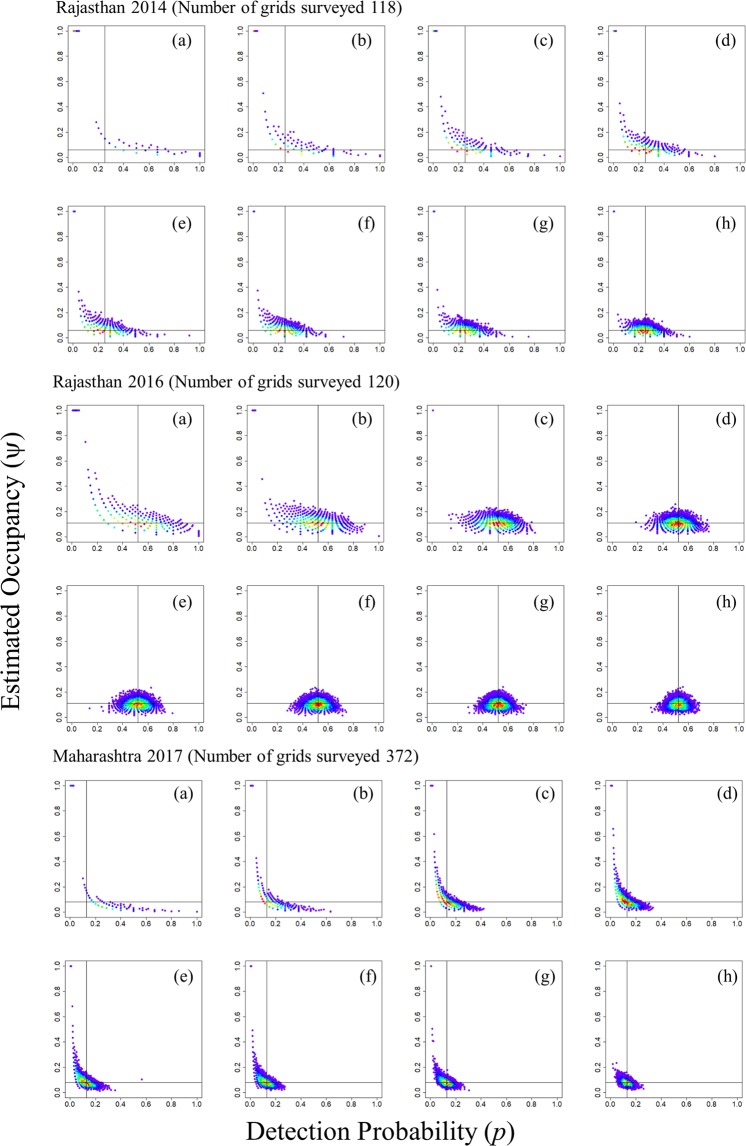
Distribution of the maximum likelihood estimates (MLE) from three landscape surveys dataset (Rajasthan 2014 ψ = 0.06, p = 0.25, 10000 iterations; Rajasthan 2016 ψ = 0.11, p = 0.52, 10000 iterations; Maharashtra 2017 ψ = 0.08, p = 0.13, 10000 iterations) with varying number of survey replicates [(**a**)2, (**b**)3, (**c**)4, (**d**)5, (**e**) 6, (**f**) 7, (**g**) 8, (**h**) 10 visits] keeping the number of survey grids constant.

Our results from cumulative detection probability suggest that a minimum of four replicates are required to reach the cumulative detection probability threshold of 0.8 (Fig. 1). Except for the Rajasthan survey of 2014, boundary estimates of the other two surveys were less than 10% with 4 sampling replicates (Table 2). The mean squared error of the occupancy and detection probability estimate with 4 replicates was also less than the usually targeted cut-off of 0.075^22^. The boundary estimates and mean squared error of both occupancy and detection probability decreased with an increase in sampling replicate. Mean squared error (MSE) of occupancy for the Rajasthan 2014 survey was 0.568 for 2 replicates and 0.0052 for 10 replicates; whereas, the MSE of occupancy for the Maharashtra 2017 survey decreased from 0.514 to 0.0004 for 2 to 10 replicates, respectively (Table 2, Fig. 2). In both surveys, the boundary values decreased from 60% to <1% as the replicates increased from 2 to 10 (Table 2, Fig. 2). Decline in MSE and boundary values indicate that more temporal replicates are required for robust assessment of occupancy of elusive and long ranging species like the GIB.

**Table 2:** Mean squared errors (MSE) associated with detection probability and occupancy, the percentage of boundary values associated with surveys carried out at Rajasthan and Maharashtra for GIB using the same protocol with a grid size of 12 × 12 km.

Number of replicates	2014 Rajasthan Survey			2016 Rajasthan Survey			2017 Maharashtra survey		
	psi MSE	p MSE	% of boundary values	psi MSE	p MSE	% of boundary values	psi MSE	p MSE	% of boundary values
2	0.5684	0.0815	60.50%	0.0233	0.0308	2.60%	0.5136	0.0242	60.60%
3	0.3045	0.0396	33.60%	0.0018	0.0123	0.10%	0.2125	0.0093	24.90%
4	0.1548	0.0228	17.10%	0.001	0.0071	0	0.0719	0.005	8.10%
5	0.077	0.0155	8.50%	0.0008	0.0048	0	0.0212	0.003	2.20%
6	0.0388	0.0111	4.20%	0.0008	0.0037	0	0.0052	0.0021	0.40%
7	0.0248	0.0085	2.70%	0.0008	0.0032	0	0.0023	0.0016	0.10%
8	0.0134	0.0068	1.40%	0.0008	0.0027	0	0.0008	0.0012	0
10	0.0052	0.0045	0.50%	0.0008	0.0021	0	0.0004	0.0008	0

**Figure 3:**
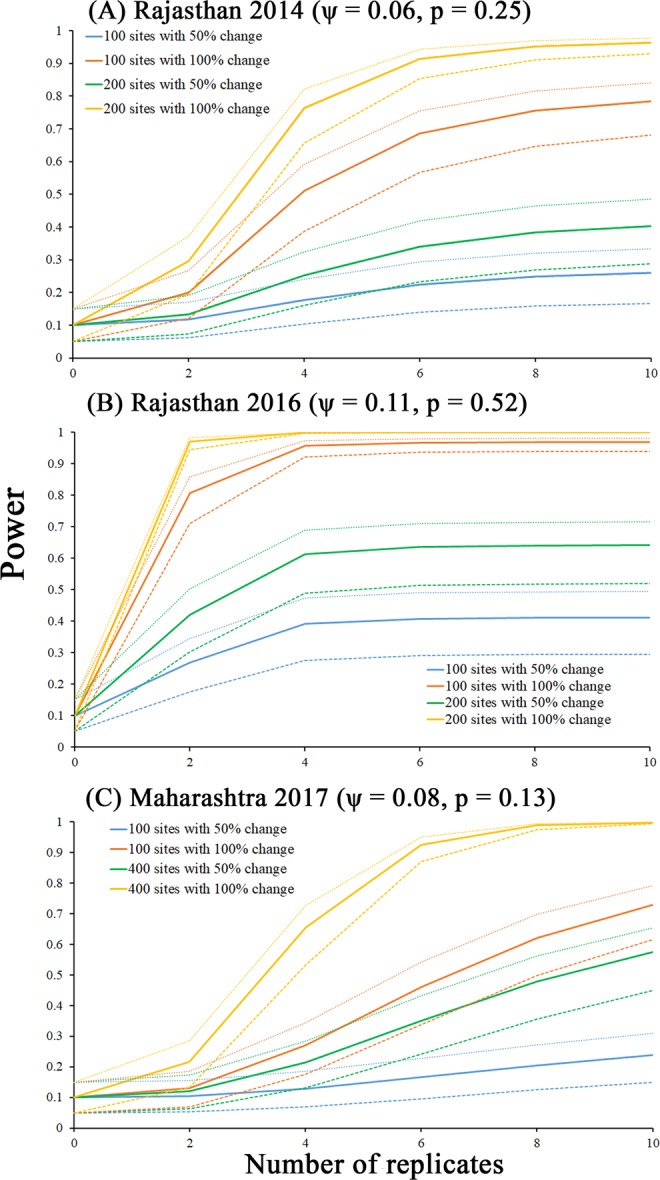
Statistical power for different surveys with different level of type-I error(α = 0.05, 0.1, 0.15) conducted across GIB distribution range (**A**) Rajasthan 2014; (**B**) Rajasthan 2016; (**C**) Maharashtra 2017; with respect to number of sampling replicates (K). We computed power for all three landscapes with four different combinations with different number of sampling sites and percent change. For each combination, the solid line represents (Type-I error)α = 0.1,the dotted line represents α = 0.15 and the dashed line represents α = 0.05.

Power analysis showed an increase in power with the number of survey replicates and sampling sites. There is >70% (78%-Rajasthan 2014, 97%-Rajasthan 2016, 73%-Maharashtra 2017) (Fig. 3) chance of detecting 100% change in occupancy with 100 sampling sites and 10 temporal replicates. The same change (76%-Rajasthan 2014, 99%-Rajasthan 2016, 70%-Maharashtra 2017) can be detected with 4–6 temporal replicates when the number of sampling sites is doubled (Fig. 3). Finally, there was insignificant change in power (mean change 0.099 ± 0.086) after 6 replicates for all surveys. The change in power was significantly more when the Type-I error was low (α = 0.05) compared to when Type-I error was 0.1 and 0.15. Also, as sampling effort (number of sites, sampling replicates) increased, there was no significant change (p _0.05,0.1_ = 0.35 and p _0.1,0.15_ = 0.40) in power even with different combinations of Type-I and Type-II error level.

## Discussion

The fragmented populations of GIBs were once distributed throughout the grasslands of northern India and the Deccan landscape in 11 states^23^, but are now confined to few fragmented populations^4^. GIBs depend on India’s semi-arid open landscape, which is rapidly being converted into agricultural lands and a significant loss in grassland habitats has been observed due to various anthropogenic factors^24^. In addition, farmers have been shifting to cash crop production from the traditional cultivation of pulses and oil seed^25,26^, which are preferred by GIB^27^. The rapid conversion of grasslands and shrublands into agricultural lands, excessive use of insecticides and pesticides, overgrazing in scrublands, increasing feral dog population, and power lines expansion are the major factors contributing to the decline of GIB population. The states of Rajasthan and Gujarat harbour more than 80% of the total GIB population; but according to a recent report, only one male GIB is left in the Kutch region of Gujarat^28^. Hence, the dwindling population of GIB needs concrete conservation measures and management policies. India has planned various site-specific management interventions such as the formation of protected areas exclusively for the species, fencing of breeding grounds to increase the success rate of breeding events, controlling free-ranging dog population, habitat improvement, as well as *ex-situ* conservation. In order to evaluate the effectiveness of such management interventions, it is of fundamental importance to evaluate any change in species occupancy with high accuracy.

The landscape level surveys along with blind test experiment, conducted by placing dummy GIBs, have revealed low detection of these birds which can be further confounded by low population size, nomadic movement and use of large agricultural landscape for foraging^12^. At least 4 temporal replicates for each grid across the landscape is required for robust estimation. Effective sampling design for species like GIB, with low occupancy estimates (6–12%) and low detection probability (0.12–0.25), needs to ensure the parameters of interest meet the design target before spending valuable time and effort in the field. We defined different thresholds for type-I error- detecting a change when there is no change (α = 0.05, 0.1, 0.15) and type-II error- not detecting a change when there is a change β = (0.7, 0.8, 0.9) for the power analysis to avoid a misleading inference about population change. Similar to other studies, we observed an increase in power with survey sites or survey replicates^21,29^. Sampling designs very often fail to provide sufficient statistical power for elusive and rare species, hence it becomes fundamental to increase sampling replicates for a robust assessment. Surveys carried out in Rajasthan during 2016 had the highest occupancy and detection probability hence had the highest power (0.958) compared to our other surveys (Fig. 3). The result reflects the need for higher sampling effort where occupancy and detection probability of GIB is lower particularly for states like Gujarat and Maharashtra. The outcome of power analysis concurred with the mean-squared error and cumulative detection probability results, which showed an optimum of four replicates for a threshold of 0.8.

For a species like GIB, it is imperative to include the precision of *p* in designing spatial and temporal replicates. For such a situation, the best protocol may require more replication than in the case where precision of the occupancy estimator is considered^30^. In this paper, we addressed the need for an innovative way to predict the change in population of an elusive and long-ranging species across the landscape. Our recommended modifications to the existing protocol will improve the precision to detect change in population if any. In comparison with distance sampling, occupancy sampling is more robust for analysis with low sample size, which is a pertinent issue while handling sparse datasets of such elusive species. The broad interval of population estimates resulting from distance sampling can pose a major issue to inferring trends and evaluating conservation strategies. Moreover, robust abundance estimation of such long-ranging nomadic species would be financially and logistically challenging using distance sampling. Hence, we argue using occupancy as a surrogate for population trend to monitor GIB population. For designing robust methods an in-depth understanding of species ecology and habitat-use pattern is must especially for areas where occupancy and detection probability is low and influenced by species activity pattern and space use. The effectiveness of conservation efforts can be assessed in-depth by focusing on issues derived from the tradeoff resulting from the allocation of survey efforts^30^. Defining the trend in species population is not only governed by statistical decisions^30^ but is also influenced by species ecology. We cannot buy more time to conserve such a critically endangered species as the GIB, which may reach a level where it would be impossible to bring back the species from local extirpation. The important contribution of our study is the development of a robust protocol to assess population trends of a highly endangered species.

## Materials and Methods

### Monitoring protocol

**Figure 4:**
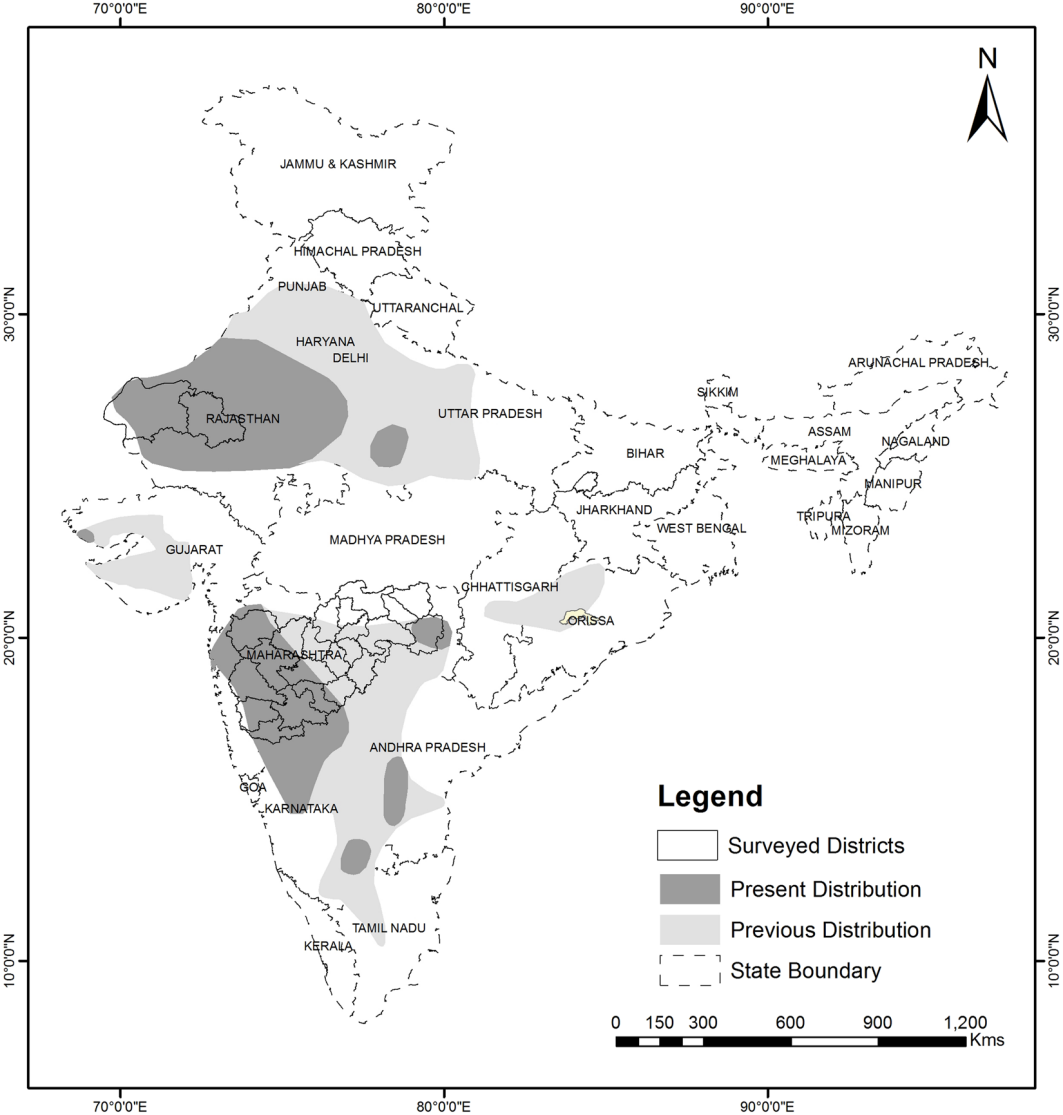
Map showing the former and present distribution of GIB in India along with the survey sites across the country (Distribution map source: BirdLife International; IUCN RedData List^3^).

Potential GIB habitat across the distribution range was divided into 12*12 km grids. Each grid was monitored simultaneously by trained survey teams to avoid detection of the same individual twice (Fig. 4). A total distance of 20 km was traversed along the trails and roads by the survey team in a slow-moving vehicle (10–20 km/hr.). For each sighting of GIB, distance from the trail, angle, GPS coordinates and number of individuals were recorded^13^.

### Blind Test to estimate detection probability

Owing to their low population density, nomadic behaviour and large range, the chances of sighting a GIB during the survey period was very low. This influences detection probability and affects occupancy estimates. In order to overcome this shortcoming, we designed a blind test using life-sized dummies of GIB to predict the probability of missing a GIB in its presence. A total of 30 GIB dummies were deployed across the survey area. These dummy GIBs were placed in areas known to be sampled by the survey team without their knowledge.

### Evaluating the effectiveness of the existing protocol

We used the detection probability from surveys conducted in the states of Rajasthan and Maharashtra to calculate the cumulative detection probability. The cumulative detection probability is p* = 1 − *(1* − *p)^k*, where k is the number of sampling replicates and p is the sampling-replicate-specific detection probability (i.e., the probability of detecting an animal during a single replicate). We estimated the optimum *k* required to surpass the threshold of 0.8, which is the probability of detecting the species at least once with 80% confidence interval^31^, in the conducted surveys.

In order to assess the effectiveness of the current protocol, we calculated the mean-squared error for both the occupancy estimate and detection probability with 10,000 simulations of the parameter estimates. We simulated capture histories with the occupancy and detection probability estimates following binomial distribution and estimated occupancy and detection probability based on the capture histories. We also evaluated boundary estimates ($\hat{\psi }=1$)^30^, which can be used to evaluate the performance of various sampling strategies to identify the optimal sampling design. Boundary values are a major issue when the number of replicates is small and the probability values are low^30^. Both of the conditions were of major concern in our case. Simulation and analysis were carried out in R 3.4 (R Core Team 2017)^32^.

Power of a statistical test given by 1 − β (Type II error) represents the probability of correctly rejecting a false hypothesis and the levels of significance indicate the relative seriousness of committing Type I and II errors^22,33^. We used power analysis to investigate robustness using detection probability and occupancy estimates of the previous surveys^22^. We considered effect size as a function of both occupancy and detection probability to calculate power. The precision of estimates usually improves with the increasing temporal replicates. However, when this is performed reducing the number of sampling sites, it creates a trade-off leading to an optimal number of repeated visits which depends on the characteristics of the species^22^.

Considering our monitoring goal with a large landscape, we assumed that making a Type II error (β) would be highly costly and chose three different levels (0.7, 0.8, 0.9) for β (i.e., not detecting a change in occupancy and detection probability when there is a change) and used three different levels (0.05, 0.1, 0.15) for α (Type I error). In this scenario, committing Type-I error (i.e. detecting a change when there is no change) would be very less feasible as the species is known for their site-fidelity. Moreover, the spatial extent of the survey encompassed all available suitable habitats of the species reducing the chances of Type I error. We tested the effectiveness by varying number of sites (100–400) and replicates (2, 4, 6, 8, 10) to investigate the power of different surveys. We constructed a power curve from the analysis keeping the estimated occupancy parameters and daily detection probability constant.
